# Definition of hemodynamic stability in blunt trauma patients: a systematic review and assessment amongst Dutch trauma team members

**DOI:** 10.1007/s00068-016-0744-8

**Published:** 2016-11-30

**Authors:** S. A. I. Loggers, T. W. A. Koedam, G. F. Giannakopoulos, E. Vandewalle, M. Erwteman, W. P. Zuidema

**Affiliations:** 1 Department of Trauma Surgery, VU University Medical Center, 7F029, De Boelelaan 1117, 1007 MB Amsterdam, The Netherlands; 2 Department of Emergency Medicine, VU University Medical Center, Amsterdam, The Netherlands; 3 Department of Anesthesiology, VU University Medical Center, Amsterdam, The Netherlands

**Keywords:** Hemodynamic instability, Hypovolemic shock, Primary survey, Blunt trauma, Triage, Trauma team

## Abstract

**Introduction:**

Trauma is a great contributor to mortality worldwide. One of the challenges in trauma care is early identification and management of bleeding. The circulatory status of blunt trauma patients in the emergency room is evaluated using hemodynamic (HD) parameters. However, there is no consensus on which parameters to use. In this study, we evaluate the used terms and definitions in the literature for HD stability and compare those to the opinion of Dutch trauma team members.

**Method:**

A systematic review was performed to collect the definitions used for HD stability. Studies describing the assessment and/or treatment of blunt trauma patients in the emergency room were included. In addition, an online survey was conducted amongst Dutch trauma team members.

**Results:**

Out of a total of 222, 67 articles were found to be eligible for inclusion. HD stability was defined in 70% of these articles. The most used parameters were systolic blood pressure and heart rate. Besides the variety of parameters, a broad range of corresponding cut-off points is noted. Despite some common ground, high inter- and intra-variability is seen for the physicians that are part of the Dutch trauma teams.

**Conclusion:**

All authors acknowledge HD stability as the most important factor in the assessment and management of blunt trauma patients. There is, however, no consensus in the literature as well as none-to-fair consensus amongst Dutch trauma team members in the definition of HD stability. A trauma team ready to co-operate with consensus-based opinions together with a valid scoring system is in our opinion the best method to assess and treat seriously injured trauma patients.

## Introduction

Trauma is a global phenomenon. In 2008, 5.1 million people (9% of total deaths) died worldwide as a result of injury. Injuries also account for 17% of the disease burden in adults aged 15–59 years in 2004 [1, 2].

Most deaths are caused by unintentional injuries including blunt trauma such as falls or road accidents. Blunt trauma accounts for an estimated 50% of the mechanism of injury proportion [1].

The assessment of the hemodynamic (HD) status in blunt trauma patients is vital for early identification and timely management of a potential hemorrhage to keep the time between injury and intervention to a minimum. In order to improve trauma care furthermore, evidence-based practice guidelines are designed and implemented in every hospital. These management schemes are often based on the presence or absence of HD stability, proposed by the American College of Surgeons Advanced Trauma Life Support (ATLS) guidelines [3].

When the patient is unstable, time is a luxury and immediate surgical intervention in combination with resuscitation is mandatory [4, 5]. When the patient is stable, more time is available for the assessment of the patient’s injuries.

Systolic blood pressure (SBP) and heart rate (HR) have traditionally been used for recognition of the shock state in ATLS and Prehospital Trauma Life Support (PTLS) guidelines [3, 6]. However, the value of these vital signs and their cut-off points have been disputed by some [7–12].

Despite the importance of the HD status of blunt trauma patients, several hemodynamic parameters [e.g., HR, respiratory rate (RR), blood pressure (BP), SPB and Revised Trauma Score (RTS)] with different cut-off points are used without general consensus about the best evidence-based practice. A combination of the traditional signs BP and HR, named Shock Index (SI) (calculated by HR/SBP), has been shown to identify beginning hemorrhage [13], need for massive transfusion [14] and predicting mortality [11, 15] more early and better than the vital signs apart.

As the initial assessment of a trauma patient concerns a multidisciplinary approach by the examining anesthesiologist, trauma surgeon and the emergency physician in the emergency room, it is important for everyone to speak the same language. Different specialities, however, bring different opinions about the best treatment if there is no clear consensus about the interpretation of all parameters. The meaning of HD instability in trauma patient is still very wide with unclear borders and lacks a clear validated definition that states which indicative parameters to use to initially assess the circulatory status.

This study assesses the definitions used for HD stability in a systematic review of the literature combined with a survey of the interpretations of HD instability in blunt trauma patients in the ER amongst Dutch trauma team members in order to establish the level of consensus about HD stability for blunt trauma patients.

## Method

### Review of the literature

A systematic search of the literature was conducted using the computerized bibliographic database MEDLINE and Embase. Both were searched for English and Dutch articles published from 2005 to 2015 concerning the diagnostics and/or treatment of adult patients suffering exclusively from blunt injury. The following combination of MeSH terms were used in the literature search: blunt trauma AND diagnostics OR management AND hemodynamic instability (full search available upon request).

**Fig. 1:**
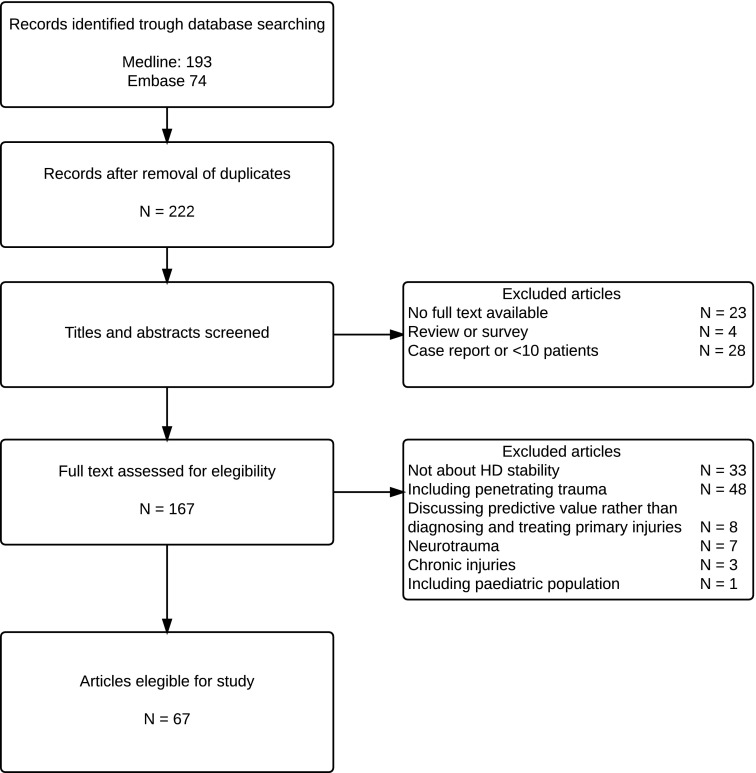
HD stability search flowchart

Studies describing patients with severe neurological injuries, septic shock and/or chronic injuries, as well as studies of which the full text article was not available or articles published in another language than English or Dutch were excluded. Studies with less than ten patients, patients aged under 16 years, reviews and studies discussing predictive values in trauma patients rather than focusing on diagnosing and treating primary injuries were also excluded (see Fig. 1). Abstracts were independently screened by two different authors for inclusion criteria. If there was disagreement between the two authors, the opinion of a third authors was decisive. After abstracts were screened for eligibility, this procedure was repeated for the full text. After studies were found to be eligible for inclusion, the hemodynamic parameters, corresponding cut-off points and timing, type and amount of fluid resuscitation used for the definition of HD stability were processed in an SPSS 22^®^ database.

### Survey amongst trauma team members

In addition to the systematic review of the literature, an internet based survey study using a questionnaire was sent to all trauma surgeons, emergency MDs and anesthesiologists in The Netherlands via email asking their opinion about which parameters to use best with their corresponding cut-off points in HD unstable patients. The questionnaire included five cases of patients suffering blunt injuries. Additional information was presented after the first assessment in order to simulate the dynamic aspect of the evaluation of these patients. Physicians who were not part of their hospital’s trauma team were excluded.

By asking the different physicians to make an assessment of the patient’s hemodynamic situation (stable/unstable) a comparison of their judgment was made by calculating the Cohen’s Kappa-value on agreement in and between these three groups. Cohen Kappa-value was defined as no agreement <0, none-to-slight agreement between 0.01 and 0.20, fair agreement between 0.21 and 0.40 and general agreement with values over 0.41. High intra-variability was defined if agreement was found between 35 and 65%. All data was processed in an SPSS 22^®^ database.

## Results

### Review

#### Demographics

Out of the 222 different papers identified, 67 were considered eligible for this study (see Fig. 1) [16–82]. Out of the final selection 52% described the diagnostic and/or treatment pathway of blunt abdominal injuries, 18% of pelvic injuries, 10% of vascular injuries, 10% of thoracic injuries, 1.5% injuries of the extremities and 8% injuries of blunt trauma patients in general.

#### Definition of hemodynamic stability

All studies acknowledged the term HD stability as a decisive factor in the management of blunt trauma patients [16–82]. However, as shown in Table 1, different combinations of parameters are used in the assessment of HD stability. In 70% (47/67) of the studies HD stability was defined. The parameters were measured either at admission (53%) or after resuscitation in the ED (30%). The time of measurement or timing of resuscitation was not defined in 17%.

**Table 1:** Top 5 used definitions of hemodynamic instability with corresponding cut-off points

	Parameters	Cut-off points	Freq.	% of studies	References
1	Only SBP	<80–100 mmHg at admission	13	27.6	[19, 22, 25, 27, 49, 59, 62, 63, 70, 73, 74, 79, 81]
2	SBP and Response to Fluid resuscitation PRBC	<90 mmHg at admission or after >1–2 L initial fluids >2–6 PRBC/24 h	12	25.5	[16, 18, 32, 34, 41, 55, 71, 75]
3	SBP and HR	<90 mmHg at admission >100–130 bpm at admission	10	21.3	[26, 29, 30, 52, 61, 64, 68, 76, 78, 82]
4	SBP and HR and Response to Fluid resuscitation	<90 mmHg at admission >100–120 bpm at admission or after >1–2 l initial fluids	4	8.5	[33, 40, 67, 80]
5	SBP and SI	<90 mmHg at admission >1 at admission	4	8.5	[39, 45, 58, 72]
	Total		43	92	43/47

Top five used definitions of hemodynamic instability in the articles, organized by the different combinations of parameters with their range of corresponding cut-off points and percentage of articles using this definition

*HR* heart rate*, SBP* systolic blood pressure, *SI* Shock Index (HF/SBP)

#### Parameters

In total nine different parameters or combinations of parameters are used to define HD stability. The most used parameter is the SBP (53.2%), measured either at admission (52%) or after fluid resuscitation (32%). The second most used parameter is a combination of SBP and HR (29.8%), measured either at admission (50%) or after fluid resuscitation (28.6%). Also SI was used in five occasions, either in combination with SBP or fluid resuscitation. No studies used HR alone to define HD stability. An overview of the used parameters for assessing HD stability is shown in Table 1.

#### Cut-off points

A wide range of cut-off points, meaning the moment when the physician interpreted the value as abnormal, is used (see Table 1). A blunt trauma patient was mainly defined as unstable if the SBP was below 90 mmHg (range 70–100 mmHg). 82% used an SBP below 90 mmHg as a marker for HD instability. A cut-off point for HR was defined by 14 articles and ranged from higher than 100 to higher than 130. HD instability was defined by a HR higher than 120 bpm in 65%, with 15% of the articles using the cut-off points of higher than 110 bpm and higher than 100 bpm. The average cutoff point for SBP was below 91 mmHg and the average cutoff point for HR higher than 116 bpm.

Another large variation in definition is whether these previously mentioned values were measured at admission or after resuscitation. 18 studies used fluid resuscitation as part of their definition, of which five authors did not define the amount of resuscitation. The amount of resuscitation was defined by either the used amount of crystalloids and/or PRBC. The amount of crystalloids ranged between 1 and 2 l. The amount of PRBC given ranged between two and six units. The cut-off point for the SI was always 1.0.

### Survey amongst Dutch trauma team members (TTM)

The response rate was 64% with an average completion time of 15 min. A total of 251 responders completed the survey. 225 (90%) of those were part of their hospital’s trauma team. 36% were emergency physicians, 35% anesthesiologists and 29% trauma surgeons.

#### Parameters

When asked what specialists would consider the most important parameter, the three traditional vital signs (HR, sSBP and RR) as their most used parameters. HR was considered the most important by all TTM in 45%, followed by the sSBP (30%) and the RR (18%). An overview of the most used parameters is shown in Table 2. The least consensus about which parameters to use best is seen in the anesthesiologists group. The emergency physicians and trauma surgeon’s groups show more consensus, with the latter group showing the biggest differences in percentages.

**Table 2:** Overview of the top three most used vital parameters amongst different physicians in the trauma bay

	Emergency physicians (*n* = 81)			Anesthesiologists (*n* = 78)			Trauma surgeons *(n* = 66)			Total (*n* = 255)		
	1st (%)	2nd (%)	3rd (%)	1st (%)	2nd (%)	3rd (%)	1st (%)	2nd (%)	3rd (%)	1st (%)	2nd (%)	3rd (%)
Most used parameter	HR 45	RR 19	sSBP 16	HR 31	sSBP 30	SI 9	HR 62	sSBP 21	pSBP 7	HR 45	sSBP 23	RR pSBP 8
2nd most used parameter	HR 33	sSBP 30	RR 17	HR 31	sSBP 23	RR BL 9	sSBP 38	HR 18	pSBP 14	sSBP 30	HR 28	RR 10
3rd most used parameter	RR 25	sSBP 16	HR pSBP 14	HR 19	sSBP 15	BL 13	RR sSBP 16	HR pSBP 10	sDBP 8	RR 18	HR sSBP 15	pSBP BL 10

The results are ranked by the most used, 2nd most used and 3rd most used vital parameter per physician. Every first, second and third most used vital parameter are also subdivided by the 1st, 2nd or 3rd most preferred parameter per physician in order to display the range of preferences. For example, 45% of the 81 emergency physicians used HR as their most important parameter

*HR* heart rate (beats per minute*), sSBP* shockroom systolic blood pressure (mmHg), *pSBP* prehospital systolic blood pressure (mmHg), *RR* respiratory rate (per minute), *sDBP* shockroom diastolic blood pressure (mmHg), *SI* Shock Index (HF/SBP), *BL* blood loss (l)

#### Cut-off points

The corresponding average cut-off points, differed between the different specialties. Purely based on the SBP, HR and RR with corresponding cut-off points, emergency physicians assess a trauma patient more early as potentially HD unstable, followed by trauma surgeons and anesthesiologists, respectively. An overview of the different cut-off points used for HD stability is shown in Table 3.

**Table 3:** Overview of top three most used parameters with their corresponding cut-off points used by Dutch trauma team members

Parameter	Cut-off point	Emergency physicians		Anesthesiologists		Trauma surgeons		Total (*n* = 85)	
		pSBP (*n* = 42)	sSBP (*n* = 61)	pSBP (*n* = 23)	sSBP (*n* = 51)	pSBP (*n* = 20)	sSBP (*n* = 44)	pSBP	sSBP
SBP (mmHg)	<110–105	7%	9%	4%	2%	5%	5%	6%	5%
	<100–95	59%	52%	46%	39%	55%	41%	56%	45%
	<90–85	23%	34%	30%	37%	25%	43%	26%	38%
	<80	10%	7%	17%	22%	15%	11%	13%	13%
	Avg. (mmHg)	<96	<95	<93	<91	<94	<94	<95	<94

Parameter	Cut-off point	Emergency physicians (*n* = 66)	Anesthesiologists (*n* = 56)	Trauma surgeons (*n* = 52)	Total (*n* = 174)
HR (bpm)	>90–95	3%	2%	6%	4%
	>100–105	75%	40%	50%	55%
	>110–115	15%	21%	23%	19%
	>120	8%	38%	29%	22%
	Avg. (bpm)	>103	>110	>107	> 106

Parameter	Cut-off point	Emergency physicians (*n* = 56)	Anesthesiologists (*n* = 39)	Trauma surgeons (*n* = 22)	Total (*n* = 117)
RR (p/m)	<40–35	4%	18%	23%	14%
	<30–25	63%	59%	50%	59%
	<20–15	31%	23%	27%	27%
	Avg. (p/m)	<34	<33	<33	< 34

*HR* heart rate (beats per minute), *SBP* systolic blood pressure (mmHg)*, RR* respiratory rate (per minute), *pSBP* prehospital systolic blood pressure (mmHg), *sSBP* shockroom systolic blood pressure (mmHg)

#### Increasing heart rate

All TTM will assess a patient with a higher heart rate more often as unstable. In the dynamic aspect of the survey, agreement is seen for all specialties when the HR is 98 bpm with an average judgment of HD instability of 13%. When the HR rises to 108 bpm, none-to-slight agreement is seen between the anesthesiologists (47%) compared to trauma surgeons (63% with *p* = 0.09) and emergency physicians (68% with 0.015), where anesthesiologists are less likely to judge a patient as HD unstable. When the HR reaches 118 bpm none-to-fair agreement is seen amongst all TTM specialists with emergency physicians (87%) judging the patient as HD unstable the most and anesthesiologists (69%) the least.

High intra-variability is seen amongst trauma surgeons (63% unstable) and anesthesiologists (47% unstable) when the HR reaches 108 bpm. No high intra-variability is seen amongst emergency physicians.

#### Decreasing blood pressure

Total consensus is reached when the BP of a young patient is 120/78 mmHg. When the BP drops to 105/67 mmHg and 95/65 mmHg, none-to-fair agreement is seen between emergency physicians (30 and 77%) compared to trauma surgeons (22% with *p* = 0.404 and 51% with *p* = 0.004) and anesthesiologists (17% with *p* = 0.104 and 56% with *p* = 0.014), in which emergency physicians are more likely to judge a patient as HD unstable. No differences in agreement are found in young patients between trauma surgeons and anesthesiologists.

When the same parameters are tested in a 65-year-old patient this changes. Elderly patients are more frequently assessed as unstable with the same blood pressure as a young patient for all specialties. When the BP of a 65 year old reaches 105/67 mmHg, anesthesiologists (31%) will judge a patient as less HD unstable compared to trauma surgeons (39% with *p* = 0.402) and emergency physicians (46% with *p* = 0.096). Agreement is seen between trauma surgeons and emergency physicians for the BP of 120/75 and 105/67 mmHg) of older patients.

High intra-variability is seen for the BP of a young patients amongst trauma surgeons (51% unstable) and anesthesiologists (56% unstable) when the BP reaches 95/65 mmHg. In older patients high intra-variability is seen amongst emergency physicians (46% unstable) and trauma surgeons (39%) when the BP reaches 105/67 mmHg.

#### Negative versus positive FAST

None-to-slight agreement is shown between trauma surgeons and the other TTM specialists with a positive result of the FAST. When the FAST shows any fluid around organs or free fluid in the abdomen, trauma surgeons (27 and 29%, respectively) are significantly less likely to judge a patient as HD unstable compared to baseline parameters than anesthesiologists (43% with *p* = 0.095 and 53% with *p* = 0.011, respectively) and emergency physicians (44% with *p* = 0.077 and 55% with *p* = 0.007). There are no significant differences between anesthesiologists and emergency physicians.

High intra-variability is seen amongst anesthesiologists and emergency physicians when the FAST shows either free fluid around a solid organ (43 and 44% unstable, respectively) or free abdominal fluid (53 and 55% unstable, respectively). No high intra-variability is seen amongst trauma surgeons.

#### Decreasing hemoglobin level

There is none-to-slight agreement between trauma surgeons and the other two specialties about the use of low hemoglobin (Hb) levels upon arrival in the trauma bay when the Hb levels are 7.2, 6.2 and 5.2 mmol/l. Trauma surgeons are significantly less likely to be influenced by the level of Hb in their decision making of HD instability compared to emergency physicians and anesthesiologists. Trauma surgeons are significantly less likely to judge a patient as HD unstable when Hb levels are 6.2 or 5.2 mmol/l in male patients compared to anesthesiologists (*p* = 0.009 and 0.014) and emergency physicians (*p* = 0.028 and 0.009). This also accounts for Hb levels of 6.2 and 5.2 mmol/l in female patients (*p* = 0.03 for anesthesiologists and *p* = 0.001 for emergency physicians). The only time where there is no agreement between anesthesiologists and emergency physicians is when the Hb levels of females reach 5.2 mmol/l.

High intra-variability is seen amongst emergency physicians when the HB levels are 6.2 mmol/l in male (51% unstable) and females (44% unstable) patients. This is also seen amongst anesthesiologists with 51 and 55% unstable for the same HB levels for males and females, respectively. High intra-variability is seen amongst trauma surgeons when the HB levels reaches 5.2 mmol/l in male (51% unstable) and female (53% unstable) patients.

#### Shock Index (SI)

The influence of Shock Index on the HD assessment in the Dutch emergency bay is low, with only 42 TTM defining a cut-off point for SI, with an average cut-off point of higher than 1.0.

#### Resuscitation

TTM (*n* = 40) on average will judge a patient as HD unstable when five or more units of packed red blood cells (PRBC) are infused with response.

#### Comparison of literature versus practice

As the review of the literature showed that the SBP, used in 53% of the definitions, measured either at admission before or after fluid resuscitation, was considered the most important parameter. No study defined HD stability purely based on the HR. A combination of SBP and HR, before or after resuscitation, was used in about 30% of the cases. Different cut-off points are used for the different parameters measured before or after different amounts of fluid resuscitation. The average cutoff point for SBP was below 91 mmHg and for HR higher than 116 bpm.

The survey showed that HR is considered the most important parameter for the assessment of HD stability by Dutch TTM in 45%, followed by SBP and RR. The average cutoff point for HR was higher than 106 bpm, for SBP below 95 mmHg.

## Discussion

During the early assessment, the trauma team needs to triage blunt trauma patients according to their HD status in order to choose the best treatment pathway determined by evidence-based research. With multiple specialties featuring in current trauma teams, a multidisciplinary approach will only benefit the treatment of a trauma patient if the interdisciplinary differences in language are settled, especially in moments when time is scarce. Our systematic review and analyses of a survey amongst Dutch TTM has showed that there is a lack of consensus about which parameters and their corresponding cut-off points to use for the judgement of HD instability. If a definition of HD instability is even given in the literature, there are clear differences in the used parameters and corresponding cut-off points. There is high inter- and intra-variability between and amongst the different specialties featuring in their trauma team. This study also shows differences in parameters used for HD stability definition between current literature and TTM in The Netherlands.

To create a uniform language, research is performed to create consensus-based guidelines with clear treatment paths for blunt trauma patients. The lack of consensus about parameters and cut-off points in literature could create difficulties in making population-based conclusions for the evidence-based practice since study groups in literature are not fully comparable.

In the attempt to make uniform policy within the trauma team consisting of a trauma surgeon, anesthesiologist and emergency physician, the evaluation of trauma patients in the Dutch emergency ward is organized according to the ATLS principles. The ATLS guideline uses the term hemorrhagic shock (often used as alternative for hemodynamic instability) based on the percentage or amount of blood loss, which would correspond with a certain increase of HR, RR and a certain decrease in SBP, urinary output and Glasgow Coma Scale. The validity of this classification is, however, under debate [7]. An online survey conducted by Mutschler et al. amongst 383 ATLS course directors and instructors confirms the doubts over the ATLS classification of shock. They showed that although the “A, B, C, D, E” approach is widely implemented, the general opinion is that only a limited number of patients can be classified by the current ATLS classification of shock. Furthermore, only 10.9% considered the ATLS classification of hypovolemic shock as a ‘good guide’ for fluid resuscitation and blood product transfusion, whereas 45.1% stated that this classification only ‘may help’ or has ‘no impact’ to guide resuscitation strategies [83].

Bland et al. already showed the difficulties in judging the HD status of critically ill patients back in 1985. They state that traditional abnormal vital parameters might not be sufficient to define HD instability. They state that even when vital signs are normal, some patients can have concealed deficiencies in tissue oxygenation [84].

Up till today HD stability is based on clinical gestalt. Clinical gestalt by itself is known to be a poor predictor for massive transfusion, or death in trauma patients, with sensitivity as low as 66% [85]. Several scoring systems have been developed to create a uniform definition for HD instability based on hemodynamic parameters and their ability to predict mortality or massive transfusion.

Meredith et al. devised the first scoring system, ‘Hemodynamic Instability Score’ (HIS), in 1994 to aid management of blunt hepatic trauma because of the large portion of unnecessary laparotomies. The scoring system was based on hypotension defined as SBP below 100 mmHg and response to initial resuscitation and need for ongoing fluid resuscitation [86]. Moore et al. [87] noted that continuing considerable variability in the definitions of HD instability and the lack of a validated scoring system. They modified the HIS by changing the cut-off point of SBP to lesser than 90 mmHg, adding tachycardia as greater than 130 bpm and response to initial advanced trauma life support recommended volume loading and the need for ongoing resuscitation including PRBC transfusion. This classification is, however, still to be validated in prospective studies.

Other, more recent, scoring systems for prediction of massive transfusion (MT), which partly includes prediction of persistent hemodynamic instability, such as the TASH [88], ABC [89] and the revised MTS score [90] use similar, or include more hemodynamic parameters as the parameters used in the HIS score. The TASH score weighs different hemodynamic parameters with several laboratory values, whereas the ABC score relies purely on hemodynamic parameters and the outcome of the FAST-echo. The revised MTS score uses only SBP as a vital parameter combined with temperature and several laboratory values based on the triangle of death in trauma patients (hypothermia, coagulopathy and metabolic acidosis).

Brockamp et al. reviewed several of these scoring systems, including the TASH and the ABC. The results, interestingly enough, showed that the only two scoring systems (TASH and PWH/Rainer) that used base deficit (BD) as a surrogate for hypoperfusion, showed the highest overall accuracy in predicting ongoing hemorrhage and MT [91].

Another study by Mutschler et al. [92], suggests the usefulness of BD in the ED. Based on a retrospective study that included over 16,000 patients from the Trauma Register DGU^®^, they proposed a shock classification based on the levels of BD on ED admission. The found that their four proposed classes of worsening of BD seems to predict transfusion requirements and mortality more significantly more accurate than the current groups in the ATLS classification. BD might be a relevant clinical approach to early risk-stratify severely injured patients in the state of hypovolemic shock and for blood product transfusion during initial assessment.

As mentioned before SI has been developed and an abnormal SI values have showed to be a better predictor for transfusion and mortality in trauma patients than the vital signs apart [11, 13–15]. Recently Joseph et al. describe a DSI (SI–ER–SI-Field) in which they showed that a positive delta SI (DSI) is a better predictor for mortality (13.3%) in trauma patients compared to mortality when patients have a normal/negative DSI (9.6%). They conclude that a DSI >0.1 is associated with a higher chance of death (hazard ratio [95% CI] = 1.36 [1.29–1.45]) [93].

As the higher prediction of mortality by the decreasing SI over time showed, it is important to realize that HD instability is difficult to assess based on a single point measurement. Clinical guidelines that use development of the vital signs over a period of time to suggest a condition of HD instability will be the more preferred option, since the effects of resuscitation will also have to be awaited.

Which baseline parameters should be used define HD instability remains a point of debate. Many articles have been published describing hemodynamic parameters and their ability to predict mortality. When reviewing several HD scoring systems in combination with our literature search, a possible modification should be proposed by adding instability measured by combination of the change in SBP and HR from the field into the ER, calculated as the DSI. Another option is to add hemoglobin levels as some authors suggest or use the indicative value of hemoglobin level at admission, a drop of hemoglobin in the emergency bay after volume therapy or inadequate increase of hemoglobin after PRBC transfusion. Also base excess should be implemented in this system as proposed by Mutschler et al. [92]. The lack of consensus about the consequences of a positive FAST on the judgement of the HD status of the patient makes that this item should be left out of a scoring system, although the FAST is a vital part in the trauma screening. This modified HD scoring system should be easy to calculate and time to obtain results should be kept to a minimum in order to quickly establish the HD status of the trauma patient. It is important to realize that these systems are indicative and will only indicate a patient at risk for HD instability. With the current lack of consensus as this study shows and with the heterogeneity of the trauma patient population, combined with the low sensitivity of the clinical gestalt, a valid scoring system should be to focus of future guidelines.

## Conclusion

Based on this review of the literature, it is clear that HD stability is acknowledged as a vital part in the management of blunt trauma patients. However, different parameters and cut-off points are used in literature and daily practice. The interpretation of the circulatory status is highly variable and depends the author’s personal choices. The same accounts for Dutch TTM. There are structural differences within and between the different physicians participating in their hospital’s trauma team in the assessment of the HD stability. We can conclude that the term HD stability is used without full general consensus. A trauma team ready to co-operate with consensus-based opinions and a clear definition of HD stability together with a valid scoring system is in our opinion the best method to assess and treat seriously injured trauma patients.
